# Absorption‐coefficient calculation of short‐wavelength photoresist materials: From EUV to BEUV and water window X‐ray

**DOI:** 10.1002/smo.20240043

**Published:** 2024-12-16

**Authors:** Yifeng Peng, Pengzhong Chen, Hao Chen, YouMing Si, Xiaojun Peng

**Affiliations:** ^1^ State Key Laboratory of Fine Chemicals Frontiers Science Center for Smart Materials School of Chemical Engineering Dalian University of Technology Dalian China; ^2^ State Key Laboratory of Fine Chemicals College of Material Science and Engineering Shenzhen University Shenzhen China

**Keywords:** beyond extreme ultraviolet, DFT, extreme ultraviolet, linear absorption coefficient, photoresist, water window X‐ray

## Abstract

In photolithography, shortening the exposure wavelength from ultraviolet to extreme ultraviolet (EUV, 13.5 nm) and soft X‐ray region in terms of beyond EUV (BEUV, 6.X nm) and water window X‐ray (WWX, 2.2–4.4 nm) is expected to further miniaturize the technology node down to sub‐5 nm level. However, the absorption ability of molecules in these ranges, especially WWX region, is unknown, which should be very important for the utilization of energy. Herein, the molar absorption cross sections of different elements at 2.4 nm of WWX were firstly calculated and compared with the wavelengths of 13.5 nm and 6.7 nm. Based on the absorption cross sections in these ranges and density estimation results from the density‐functional theory calculation, the linear absorption coefficients of typical resist materials, including metal‐oxy clusters, organic small molecules, polymers, and photoacid generators (PAGs), are evaluated. The analysis suggests that the Zn cluster has higher absorption in BEUV, whereas the Sn cluster has higher absorption in WWX. Doping PAGs with high EUV absorption atoms improves chemically amplified photoresist (CAR) polymer absorption performance. However, for WWX, it is necessary to introduce an absorption layer containing high WWX absorption elements such as Zr, Sn, and Hf to increase the WWX absorption.

## Graphical abstract

In photolithography, shortening the exposure wavelength from ultraviolet to extreme ultraviolet (EUV, 13.5 nm) and soft X‐ray region in terms of beyond EUV (BEUV, 6.X nm) and water window X‐ray (WWX, 2.2–4.4 nm) is expected to further miniaturize the technology node down to sub‐5 nm level. However, the absorption ability of molecules in these ranges, especially WWX region, is unknown, which should be very important for the utilization of energy. Herein, the molar absorption cross sections of different elements at 2.4 nm of WWX were firstly calculated and compared with the wavelengths of 13.5 nm and 6.7 nm. Based on the absorption cross sections in these ranges and density estimation results from the density‐functional theory calculation, the linear absorption coefficients of typical resist materials, including metal‐oxy clusters, organic small molecules, polymers, and photoacid generators (PAG), are evaluated. The analysis suggests that the Zn cluster has higher absorption in BEUV, while Sn cluster has higher absorption in WWX. Doping PAGs with high EUV absorption atoms improves chemically amplified photoresist (CAR) polymer absorption performance. However, for WWX, it is necessary to introduce an absorption layer containing high WWX absorption elements such as Zr, Sn, and Hf to increase the WWX absorption.

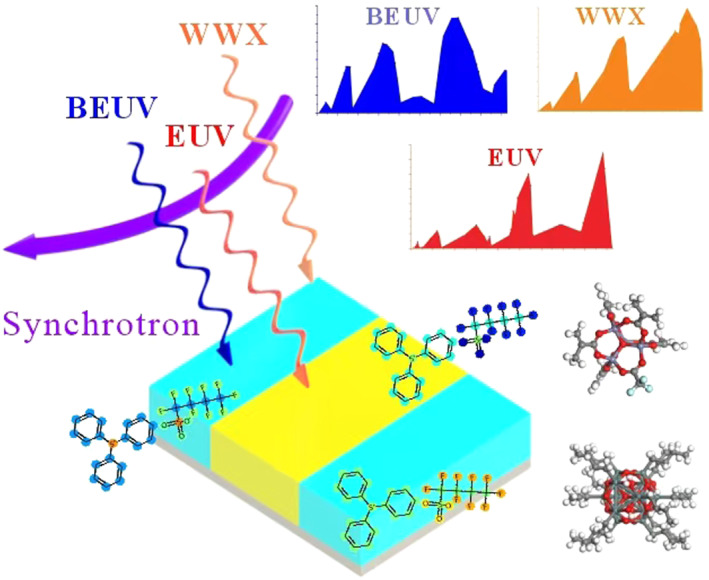

## 1 INTRODUCTION

In the semiconductor industry, extreme ultraviolet (EUV) lithography is the current edge‐cutting approach for generating sub‐10 nm technology nodes of integrated circuits. Photoresist materials capable of patterning high‐resolution characteristics are also highly required, which has deserved much attention during the last decades.^[1]^ Metal‐oxo cluster photoresists and new polymer photoresists have shown excellent performance in short‐wavelength lithography. The performance and EUV absorption rate of metal‐oxo photoresists can be optimized by adjusting the ligands.^[2]^ Meanwhile, the successful implementation of EUV lithography (EUVL) would inspire the exploration of technological innovations, for example, shortening the exposure wavelength from EUV (13.5 nm) to the soft X‐ray region in terms of beyond EUV (BEUV 6.X nm), and water window X‐ray (WWX 2.2–4.4 nm). BEUV lithography should be expected to further miniaturize the technology node down to sub‐5 nm level.^[3]^ Synchrotron sources and free‐electron laser (FEL) devices enable the platforms of BEUV and WWX lithography (WWXL) for their wide spectral range from soft X‐ray region to EUV.^[4]^ The demand for the analysis of biological materials has propelled the advancement of WWX optical apparatus,[^5^, ^6^] which makes it possible to perform WWX lithography on resist materials.^[7]^ Moreover, it has been found that the source power system transmission efficiency for the 6.X nm and 2.2–4.4 nm based on synchrotron sources and FEL is comparable and even higher than that of EUV 13.5 nm. Therefore, BEUV and WWX demonstrate their feasibility as the future generation of lithographic technology for high‐volume manufacturing of semiconductors.^[8]^

Absorption cross‐sections of EUV 13.5 nm and BEUV 6.7 nm have been calculated in several articles.[^9^, ^10^] However, there is a lack of a clear comparative evaluation of molar absorption cross sections for potential wavelength nodes such as WWX. At present, soft X‐ray lithography employs materials such as hydrogen silsesquioxane resin (HSQ) and poly(methyl methacrylate) (PMMA) as resists despite their low absorption in the short‐wavelength region.^[3]^ Recent years have witnessed the development of smart materials or stimuli‐responsive materials, which has led to a more complex structure of potential photoresist molecules.[^11^, ^12^, ^13^, ^14^] To guide the design of short‐wavelength photoresist materials and predict their performance under different synchrotron output conditions, it is necessary to determine the molar absorption cross‐section of different elements. Herein, the density‐functional theory (DFT) calculations and computational methods are firstly used to evaluate the WWX absorption behavior of various model photoresists and compare with EUV and BEUV.

## 2 MATERIALS AND METHODS

### 2.1 Materials

**FIGURE 1:**
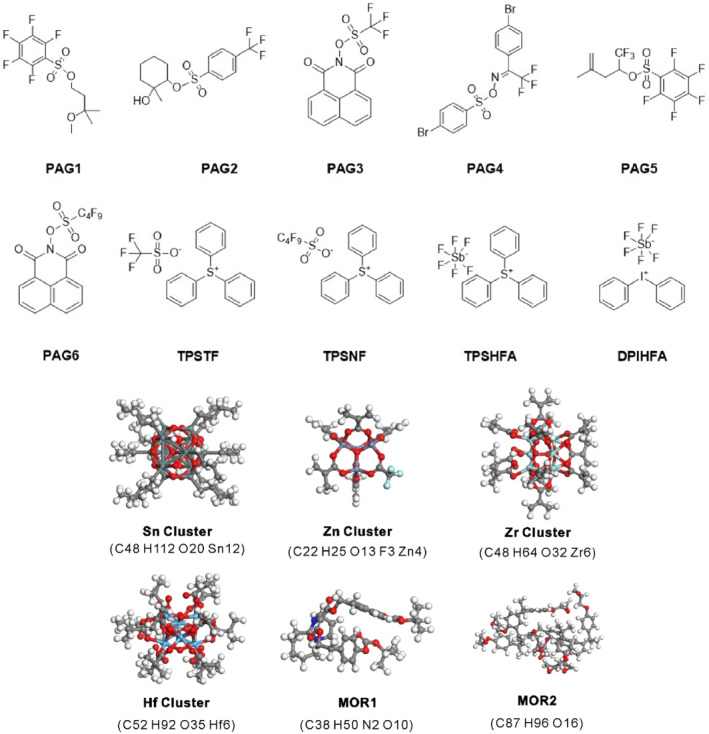
Chemical structures of typical PAGs, clusters, and molecule photoresists.

Model photoresists such as PMMA, xMT (MOR1), SU‐8 (MOR2), and HSQ resin are used in advanced research on short‐wavelength lithography. We collected the chemical structures and additional information of metal‐oxo clusters, polymers, and PAG molecules of typical photoresist systems from different references. Figure 1 displays the chemical structures of typical photo acid generators (PAG), clusters, and molecule photoresists. The density details and references for the evaluated materials are shown in Table 1.

**TABLE 1:** The density‐functional theory (DFT) basis set and density for evaluated clusters, molecule resists, polymers, and PAGs.

Name	DFT basis set	Type	Density/[g/cm^3^]	References
Sn cluster	B3LYP/LanL2DZ	Metal‐oxo cluster	2.44^a^	[15]
Zn cluster	B3LYP/LanL2DZ	Metal‐oxo cluster	1.97^a^	[16]
Hf cluster	B3LYP/LanL2DZ	Metal‐oxo cluster	2.45^a^	[17]
Zr cluster	B3LYP/LanL2DZ	Metal‐oxo cluster	1.85^a^	[18]
MOR1	B3LYP/6‐311G(d)	Molecule resist	1.36^a^	[19]
MOR2	B3LYP/6‐311G(d)	Molecule resist	1.35^a^	[20]
HSQ resin	‐‐‐	Resin	0.83	[21]
Poly(methyl methacrylate)	‐‐‐	Polymer	1.18	[22]
Polystyrene	‐‐‐	Polymer	1.00	[22]
Poly(4‐hydroxystyrene)	‐‐‐	Polymer	1.16	[22]
Poly(4‐methylstyrene)	‐‐‐	Polymer	1.04	[22]
Poly(4‐chlorostyrene)	‐‐‐	Polymer	1.22	[22]
Poly(4‐cyanostyrene)	‐‐‐	Polymer	1.07	[22]
Poly(pentafluorostyrene)	‐‐‐	Polymer	1.41	[22]
Poly(4‐methoxystyrene)	‐‐‐	Polymer	0.96	[22]
Poly(4‐acetoxystyrene)	‐‐‐	Polymer	1.06	[22]
PAG1	B3LYP/6‐311G(d)	Non‐ionic PAG	1.73^a^	[23]
PAG2	B3LYP/6‐311G(d)	Non‐ionic PAG	1.55^a^	[24]
PAG3	B3LYP/6‐311G(d)	Non‐ionic PAG	1.77^a^	[25]
PAG4	B3LYP/6‐311G(d)	Non‐ionic PAG	1.88^a^	[26]
PAG5	B3LYP/6‐311G(d)	Non‐ionic PAG	1.93^a^	[23]
PAG6	B3LYP/6‐311G(d)	Non‐ionic PAG	1.99^a^	[25]
DPIHFA	B3LYP/LanL2DZ	Ionic PAG	2.30^a^	[27]
TPSHFA	B3LYP/LanL2DZ	Ionic PAG	1.93^a^	[27]
TPSNF	B3LYP/6‐311G(d)	Ionic PAG	1.70^a^	[27]
TPSTF	B3LYP/6‐311G(d)	Ionic PAG	1.56^a^	[27]

^a^ Estimated from DFT calculation.

### 2.2 Methods for molar absorption cross section

Based on the experimental dataset,^[28]^ the molar absorption with different elements for EUV, BEUV, and WWX photons was calculated using linear interpolation. The formula for molar absorption cross‐section is presented in Equation (1).

(1) $$\begin{array}{c} \Omega_{i}=\left(\alpha \mu \left(E_{1}\right)+\beta \mu \left(E_{2}\right)\right)\varepsilon_{i}N_{A}\\ \alpha =\frac{E-E_{2}}{E_{1}-E_{2}},\beta =\frac{E-E_{1}}{E_{2}-E_{1}},E=\frac{hc}{\lambda } \end{array}$$

The symbols and units used in Equation (1) are listed as follows: Ω*_i_*—molar absorption cross‐section [cm^2^·mol^−1^]; *α*,*β*—linear interpolation coefficients; *ε_i_*—conversion factor [barns·g·atom^−1^]; μ—absorption cross‐sectional area [barns·g]; N_A_—Avogadro constant [mol^−1^]; E—photon energy [*eV*]; λ—photon wavelength [*nm*]; h—Planck constant [J · s]; and c—speed of light [m · s^−1^].

As shown in Equation (1), two experimental data points closest to the corresponding photon energy are given weight coefficients *α* and *β* according to the linear interpolation formula. The approximated absorption values are multiplied by the relevant conversion coefficients to obtain the molar absorption area of the corresponding photons. The coefficients and details are shown in Table S1 in ESI.

### 2.3 Methods for absorption coefficient of molecules and clusters

To compare the absorptivity of different molecules and cluster structures, linear absorption coefficients were calculated by Equation (2).^[29]^

(2) $$\alpha =\frac{N_{A}\sum_{i=1}^{n}c_{i}\sigma_{i}}{\text{MW}}\rho$$

*N*_A_ is the Avogadro number, MW is the molecular weight of the compound, *n* is the number of elements in the compound, *c_i_* is the relative amount of the i'th element, *σ_i_* is the absorption cross‐section of the i'th element, and *ρ* is the density (Table 1) calculated from the DFT result. The density data of clusters and molecules were computed from the molecular volume Gaussian 16 output files using Python scripts. For polymers, the density data was obtained from references.

### 2.4 DFT calculation

Quantum‐chemical calculations were performed using the Gaussian16^[30]^ program. Geometry optimizations and energy calculation for PAG1∼PAG6, MOR1, MOR2, triphenylsulfonium triflate (TPSTF), and triphenylsulfonium nonaflate (TPSNF) were carried out at the B3LYP/6‐311G(d) level. For cluster structures, triphenylsulfonium hexafluoroarsenate (TPSHFA) and diphenyliodonium hexafluoroarsenate (DPIHFA), the calculation was carried out at the B3LYP/LanL2DZ level. Absorption analysis was carried out with Python algorithms and RDKit^[31]^ library.

## 3 RESULT AND DISCUSSION

### 3.1 Molar absorption cross section of different elements for EUV, BEUV and WWX

From the view of photoresist, the response to EUV, BEUV, and WWX illumination shows similar radiation chemistry, where the secondary electrons generated by ionization of inner electrons induce molecular bond‐scission, ion detachment and the subsequent solubility‐switching reactions. The photoresist materials showing high sensitivity towards exposure light is an essential prerequisite for the radiation reactions, which also affects other factors, that is, resolution and line edge roughness. It is an efficient way to enhance the sensitivity of photoresist by introducing atoms with high absorption cross‐section into its molecular backbone. However, the sensitivity of photoresist materials depends on the wavelength of exposure because of the existence of atomic absorption edges. Therefore, investigation of the absorption coefficients of different elements at different wavelengths including 13.5 nm, 6.X nm, and 2.4 nm is beneficial for rationally designing new photoresist materials with high sensitivity as well as predicting their performance in the next generation of lithography.

**FIGURE 2:**
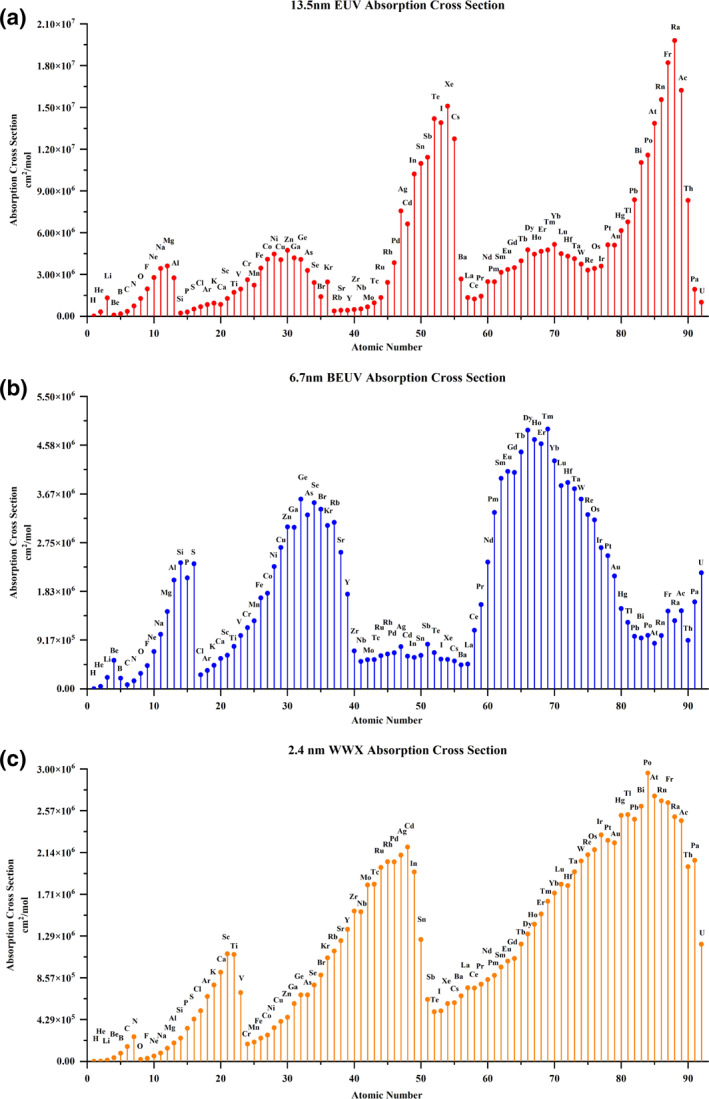
Absorption cross sections of different atoms. (a) At 13.5 nm (EUV); (b) 6.7 nm (BEUV); (c) 2.4 nm (WWX).

The absorption cross sections of different atoms for EUV, BEUV, and WWX are shown in Figure 2. The peak elements are different with wavelengths, such as, F∼Al (*Z* = 9–13), Fe∼As (*Z* = 26–33), and Ag∼I (*Z* = 47–53) with high EUV absorption cross‐section. Correspondingly, Li∼B (*Z* = 3–5), Al∼S (*Z* = 13–16), Ni∼Y (*Z* = 28–39), and Nd∼Au (*Z* = 60–79) showed high BEUV absorption. The calculated results of EUV absorption molar cross sections are consistent with those reported by Fallica et al.^[29]^ Introducing those atoms into the photoresist systems of the corresponding wavelengths can essentially improve the photon absorption performance, such as F, Zn, Sn, Sb, Te, and I for EUV and Si, P, S, Hf, Se, and Br for BEUV.

It is the first time to calculate the WWX photon molar absorption cross‐section for different atoms. C, N, Cl∼V (*Z* = 17–23), Sr∼Sn (*Z* = 38–50), and Ho∼Bi (*Z* = 67–83) are the atoms suitable for WWX (at 2.4 nm) application with large absorption cross sections. The O atom has almost no WWX photon absorption due to the water window effect, whereas nitrogen displays relatively high WWX photon absorption. Based on the preceding analysis, incorporating transducing agents containing N atoms such as triazine into the host material of the photoresist system could be a potential approach for improving the utilization of WWX photons.

### 3.2 Linear absorption coefficients of PAG and model photoresists

**FIGURE 3:**
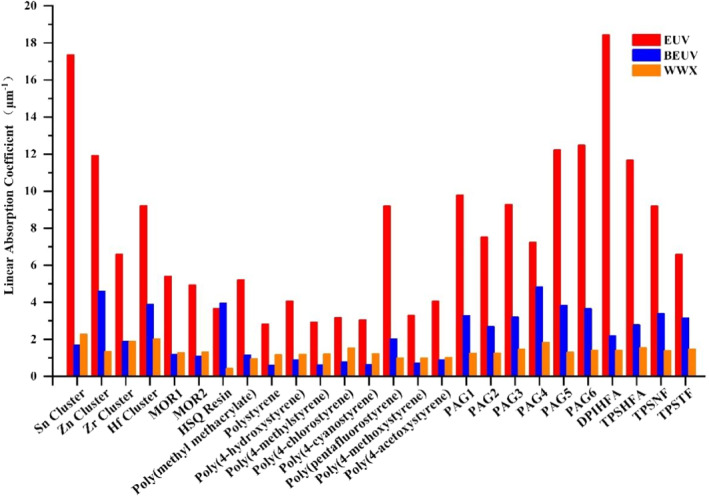
Linear absorption coefficients of different polymers, clusters and PAG molecules.

The linear absorption coefficients of different polymers, clusters, and photo acid generator (PAG) molecules are shown in Figure 3. The calculation is consistent with the previous reports on the theoretical estimation and experimental detections of the absorption cross sections in EUVs. Among the four typical metal‐oxo clusters evaluated in this study, the Sn cluster has the highest EUV absorption coefficient. The measured value of the EUV absorption coefficient of Sn clusters is within the range of 12 to 15 μm^−1^,^[29]^ similar to DFT estimated value of 17.35 μm^−1^. The estimated value for the EUV absorption coefficient of Zr clusters is 6.6 μm^−1^, with the measured values ranging from 5 to 7 μm^−1^.^[29]^ The reason for this discrepancy might be that the packing density of cluster films is reduced in comparison to the density estimated by DFT for a single cluster excluding anions.

Polymer materials that contain C, O, and H are between 3 and 5 μm^−1^. The poly(pentafluorostyrene) exhibits a higher EUV absorption coefficient of 9.2 μm^−1^ compared to the other polymers, and diphenyliodonium hexafluoroarsenate (DPIHFA, containing SbF_6_^−^ and iodonium) has the highest EUV absorption coefficient (18.4 μm^−1^) in all the PAGs evaluated in this study. Doping PAGs with “high EUV absorption” atoms therefore improves the absorption performance of polymers and chemically amplified photoresist (CAR) systems composed of polymers and PAGs.^[2]^ As the DFT calculations were performed on a single molecule, the actual packing of PAG molecules will produce a lower linear absorption coefficient than the calculated values.

As there is little report about the calculation and experimental detection of absorption cross sections of BEUV (at 6.7 nm) and WWX (at 2.4 nm), the calculation is just for prediction. Clusters with special heavy atoms such as Sn Cluster, Zr Cluster, and Hf Cluster are calculated to have high WWX absorption. The absorption coefficients of Hf cluster, Zn cluster, and hydrogen silsesquioxane resin (HSQ) resin are comparatively high for BEUV. CAR composed of hydrocarbon polymers are predicted to have limited BEUV absorption efficacy. Polymers with a lower O content, such as polystyrene and poly(4‐methylstyrene), exhibit greater absorption of WWX compared to BEUV. The difference in absorption between polymers and PAGs gradually decreases from EUV to WWX. Increased PAG absorption struggles hardly to improve photoresist absorption at low concentrations unless there is a significant improvement in the absorption factors of PAG above polymers. As the absorption coefficients of polymer materials and PAGs are nearly identical, the strategy of designing photoresists by increasing PAG absorption is predicted to be unfeasible in WWX.

**FIGURE 4:**
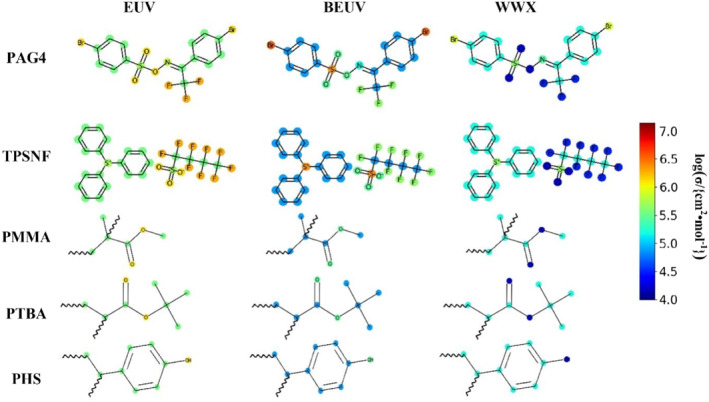
Absorption analysis of molecules under EUV, BEUV, and WWX.

The atom absorption cross sections in PAG4, TPSNF, PMMA, poly (tert‐butyl acrylate) (PTBA) and poly(p‐hydroxystyrene) (PHS) are shown in Figure 4, the photon absorption cross sections of the reddish‐colored atoms are bigger than those of the bluer‐colored atoms. The absorption centers of PAG4 are different according to the exposure wavelengths. F, Br, O, and S contribute to the absorption performance of photoresists (EUV and BEUV) while forming the source of secondary electron production. As shown in the scheme, this effect will be weakened with WWX exposure. The fluorosulfonyl group is used in TPSNF to increase the EUV absorption of PAG, but introducing F atoms has no significant effect on the WWX absorption of PAG. Consequently, different design strategies must be utilized when searching for WWX photoresist materials opposed to EUV and BEUV resists. Deposition of an absorption layer composed of a metal oxide with high WWX absorption on polymer resists, such as ZrO_2_, HfO_2_, etc., is regarded as a prospective design for WWX polymer resists.

## 4 CONCLUSION

In this paper, the molar absorption cross sections of elements *Z* = 1–92 were calculated for EUV (13.5 nm), BEUV (6.7 nm) and WWX (2.4 nm). This calculation offers a reference for short‐wavelength photoresist design. The molar absorption cross‐sections of different elements at 2.4 nm of WWX were firstly calculated and compared with the wavelengths of 13.5 nm and 6.7 nm. Based on this result, the linear absorption coefficients of different short‐wavelength model photoresist materials and PAGs were calculated. The poly(pentafluorostyrene) exhibits a higher EUV absorption coefficient than other polymers evaluated in this work. Doping PAGs with “high EUV absorption” atoms improves the absorption performance of CAR polymers. However, such an approach may not work for WWX. The low absorption of WWX photoresist could be solved by introducing top absorption atoms such as Zr, In and Hf. The analysis indicates that it is necessary to introduce an absorption layer to enhance the utilization of WWX photons for WWX polymer resists.

## Supporting information

Supporting Information S1

Supporting Information S2

Supporting Information S3

Supporting Information S4

Supporting Information S5

Supporting Information S6

Supporting Information S7

Supporting Information S8

Supporting Information S9

Supporting Information S10

Supporting Information S11

Supporting Information S12

Supporting Information S13

Supporting Information S14

Supporting Information S15

Supporting Information S16

Supporting Information S17

## Data Availability

The research data are not found.
