# Supplementary material for: Absorption‐coefficient calculation of short‐wavelength photoresist materials: From EUV to BEUV and water window X‐ray
Source: Smart Mol. 2024 Dec 16;3(4):e20240043. doi: 10.1002/smo.20240043 (PMC12755219; doi:10.1002/smo.20240043)
Supplement: Supplementary file 1 — Supporting Information S1 [file SMO2-3-e20240043-s003.docx]

## Supporting Information

1. **Numerical details of parameters**

**Table S1.** Data indexes and linear interpolation parameters.

| Photon type | Wavelength/  [nm] | Energy/  [eV]’ | E_1_/  [eV] | E_2_/  [eV] | α | β |
| --- | --- | --- | --- | --- | --- | --- |
| EUV | 13.5 | 91.8 | 91.5 | 108.5 | 0.98 | 0.18 |
| BEUV | 6.7 | 185.1 | 183.3 | 192.6 | 0.81 | 0.19 |
| SOFT-X | 2.4 | 516.6 | 511.3 | 524.9 | 0.61 | 0.39 |

As shown in **Equation 1** and **Table S1**, two experimental data points closest to the corresponding photon energy were given weight coefficients α and β according to the linear interpolation formula. The approximated absorption values were multiplied by the relevant conversion coefficients to obtain the molar absorption area of corresponding photons.

1. **Numerical Absorption Cross Section Data**

**Table S2. Absorption Cross Section**

| Atom number | EUV/[cm^2^/mol] | BEUV/[cm^2^/mol] | WWX/[cm^2^/mol] | Atom |
| --- | --- | --- | --- | --- |
| 1 | 1.516E+04 | 1.691E+03 | 6.082E+01 | H |
| 2 | 3.173E+05 | 4.491E+04 | 1.917E+03 | He |
| 3 | 1.315E+06 | 2.136E+05 | 1.166E+04 | Li |
| 4 | 8.413E+04 | 5.353E+05 | 3.699E+04 | Be |
| 5 | 1.751E+05 | 1.997E+05 | 8.366E+04 | B |
| 6 | 3.511E+05 | 7.513E+04 | 1.512E+05 | C |
| 7 | 7.323E+05 | 1.493E+05 | 2.517E+05 | N |
| 8 | 1.268E+06 | 2.885E+05 | 1.998E+04 | O |
| 9 | 1.964E+06 | 4.338E+05 | 3.122E+04 | F |
| 10 | 2.772E+06 | 7.007E+05 | 5.440E+04 | Ne |
| 11 | 3.434E+06 | 1.025E+06 | 8.444E+04 | Na |
| 12 | 3.612E+06 | 1.454E+06 | 1.337E+05 | Mg |
| 13 | 2.757E+06 | 2.044E+06 | 1.888E+05 | Al |
| 14 | 2.297E+05 | 2.372E+06 | 2.374E+05 | Si |
| 15 | 3.083E+05 | 2.084E+06 | 3.384E+05 | P |
| 16 | 5.173E+05 | 2.350E+06 | 4.325E+05 | S |
| 17 | 6.786E+05 | 2.633E+05 | 5.193E+05 | Cl |
| 18 | 8.330E+05 | 3.460E+05 | 6.652E+05 | Ar |
| Atom number | EUV/ [cm^2^/mol] | BEUV/[cm^2^/mol] | WWX/[cm^2^/mol] | Atom |
| 19 | 9.362E+05 | 4.399E+05 | 7.833E+05 | K |
| 20 | 8.507E+05 | 5.681E+05 | 9.135E+05 | Ca |
| 21 | 1.274E+06 | 6.299E+05 | 1.102E+06 | Sc |
| 22 | 1.719E+06 | 7.963E+05 | 1.096E+06 | Ti |
| 23 | 1.951E+06 | 1.001E+06 | 7.052E+05 | V |
| 24 | 2.622E+06 | 1.149E+06 | 1.768E+05 | Cr |
| 25 | 2.230E+06 | 1.277E+06 | 1.970E+05 | Mn |
| 26 | 3.448E+06 | 1.710E+06 | 2.373E+05 | Fe |
| 27 | 4.084E+06 | 1.797E+06 | 2.689E+05 | Co |
| 28 | 4.471E+06 | 2.299E+06 | 3.446E+05 | Ni |
| 29 | 4.052E+06 | 2.658E+06 | 4.095E+05 | Cu |
| 30 | 4.735E+06 | 3.045E+06 | 4.528E+05 | Zn |
| 31 | 4.184E+06 | 3.038E+06 | 5.924E+05 | Ga |
| 32 | 4.075E+06 | 3.566E+06 | 6.803E+05 | Ge |
| 33 | 3.286E+06 | 3.271E+06 | 6.813E+05 | As |
| 34 | 2.415E+06 | 3.502E+06 | 7.831E+05 | Se |
| 35 | 1.407E+06 | 3.380E+06 | 8.843E+05 | Br |
| 36 | 2.458E+06 | 3.074E+06 | 1.061E+06 | Kr |
| 37 | 3.831E+05 | 3.132E+06 | 1.130E+06 | Rb |
| 38 | 4.293E+05 | 2.567E+06 | 1.238E+06 | Sr |
| 39 | 4.235E+05 | 1.779E+06 | 1.354E+06 | Y |
| 40 | 4.866E+05 | 7.130E+05 | 1.542E+06 | Zr |
| 41 | 5.298E+05 | 5.128E+05 | 1.533E+06 | Nb |
| 42 | 6.693E+05 | 5.458E+05 | 1.810E+06 | Mo |
| 43 | 9.586E+05 | 5.475E+05 | 1.817E+06 | Tc |
| 44 | 1.339E+06 | 6.206E+05 | 1.989E+06 | Ru |
| 45 | 2.428E+06 | 6.531E+05 | 2.048E+06 | Rh |
| 46 | 3.846E+06 | 6.760E+05 | 2.046E+06 | Pd |
| 47 | 7.561E+06 | 7.900E+05 | 2.117E+06 | Ag |
| 48 | 6.628E+06 | 6.130E+05 | 2.200E+06 | Cd |
| 49 | 1.022E+07 | 5.894E+05 | 1.942E+06 | In |
| 50 | 1.098E+07 | 6.279E+05 | 1.248E+06 | Sn |
| 51 | 1.141E+07 | 8.382E+05 | 6.346E+05 | Sb |
| 52 | 1.419E+07 | 6.809E+05 | 5.082E+05 | Te |
| 53 | 1.390E+07 | 5.576E+05 | 5.178E+05 | I |
| 54 | 1.509E+07 | 5.507E+05 | 5.921E+05 | Xe |
| 55 | 1.275E+07 | 5.224E+05 | 6.023E+05 | Cs |
| 56 | 2.676E+06 | 4.484E+05 | 6.712E+05 | Ba |
| 57 | 1.335E+06 | 4.642E+05 | 7.535E+05 | La |
| 58 | 1.238E+06 | 1.099E+06 | 7.516E+05 | Ce |
| 59 | 1.443E+06 | 1.583E+06 | 7.905E+05 | Pr |
| 60 | 2.484E+06 | 2.382E+06 | 8.377E+05 | Nd |
| Atom number | EUV/ [cm^2^/mol] | BEUV/[cm^2^/mol] | WWX/[cm^2^/mol] | Atom |
| 61 | 2.471E+06 | 3.318E+06 | 8.818E+05 | Pm |
| 62 | 3.152E+06 | 3.958E+06 | 9.675E+05 | Sm |
| 63 | 3.355E+06 | 4.090E+06 | 1.028E+06 | Eu |
| 64 | 3.482E+06 | 4.069E+06 | 1.055E+06 | Gd |
| 65 | 3.979E+06 | 4.453E+06 | 1.202E+06 | Tb |
| 66 | 4.766E+06 | 4.865E+06 | 1.305E+06 | Dy |
| 67 | 4.459E+06 | 4.686E+06 | 1.408E+06 | Ho |
| 68 | 4.657E+06 | 4.610E+06 | 1.511E+06 | Er |
| 69 | 4.757E+06 | 4.887E+06 | 1.643E+06 | Tm |
| 70 | 5.152E+06 | 4.289E+06 | 1.727E+06 | Yb |
| 71 | 4.488E+06 | 3.822E+06 | 1.817E+06 | Lu |
| 72 | 4.316E+06 | 3.881E+06 | 1.803E+06 | Hf |
| 73 | 4.137E+06 | 3.764E+06 | 1.944E+06 | Ta |
| 74 | 3.742E+06 | 3.567E+06 | 2.056E+06 | W |
| 75 | 3.306E+06 | 3.277E+06 | 2.120E+06 | Re |
| 76 | 3.426E+06 | 3.176E+06 | 2.173E+06 | Os |
| 77 | 3.603E+06 | 2.655E+06 | 2.323E+06 | Ir |
| 78 | 5.124E+06 | 2.503E+06 | 2.267E+06 | Pt |
| 79 | 5.102E+06 | 2.121E+06 | 2.242E+06 | Au |
| 80 | 6.153E+06 | 1.509E+06 | 2.524E+06 | Hg |
| 81 | 6.773E+06 | 1.249E+06 | 2.531E+06 | Tl |
| 82 | 8.360E+06 | 9.851E+05 | 2.483E+06 | Pb |
| 83 | 1.104E+07 | 9.540E+05 | 2.617E+06 | Bi |
| 84 | 1.158E+07 | 1.004E+06 | 2.957E+06 | Po |
| 85 | 1.385E+07 | 8.539E+05 | 2.722E+06 | At |
| 86 | 1.555E+07 | 1.002E+06 | 2.673E+06 | Rn |
| 87 | 1.821E+07 | 1.460E+06 | 2.654E+06 | Fr |
| 88 | 1.981E+07 | 1.281E+06 | 2.509E+06 | Ra |
| 89 | 1.622E+07 | 1.466E+06 | 2.470E+06 | Ac |
| 90 | 8.315E+06 | 9.099E+05 | 1.997E+06 | Th |
| 91 | 1.931E+06 | 1.633E+06 | 2.063E+06 | Pa |
| 92 | 1.001E+06 | 2.182E+06 | 1.201E+06 | U |

1. **Metal-oxo Cluster Absorption Cross Section**


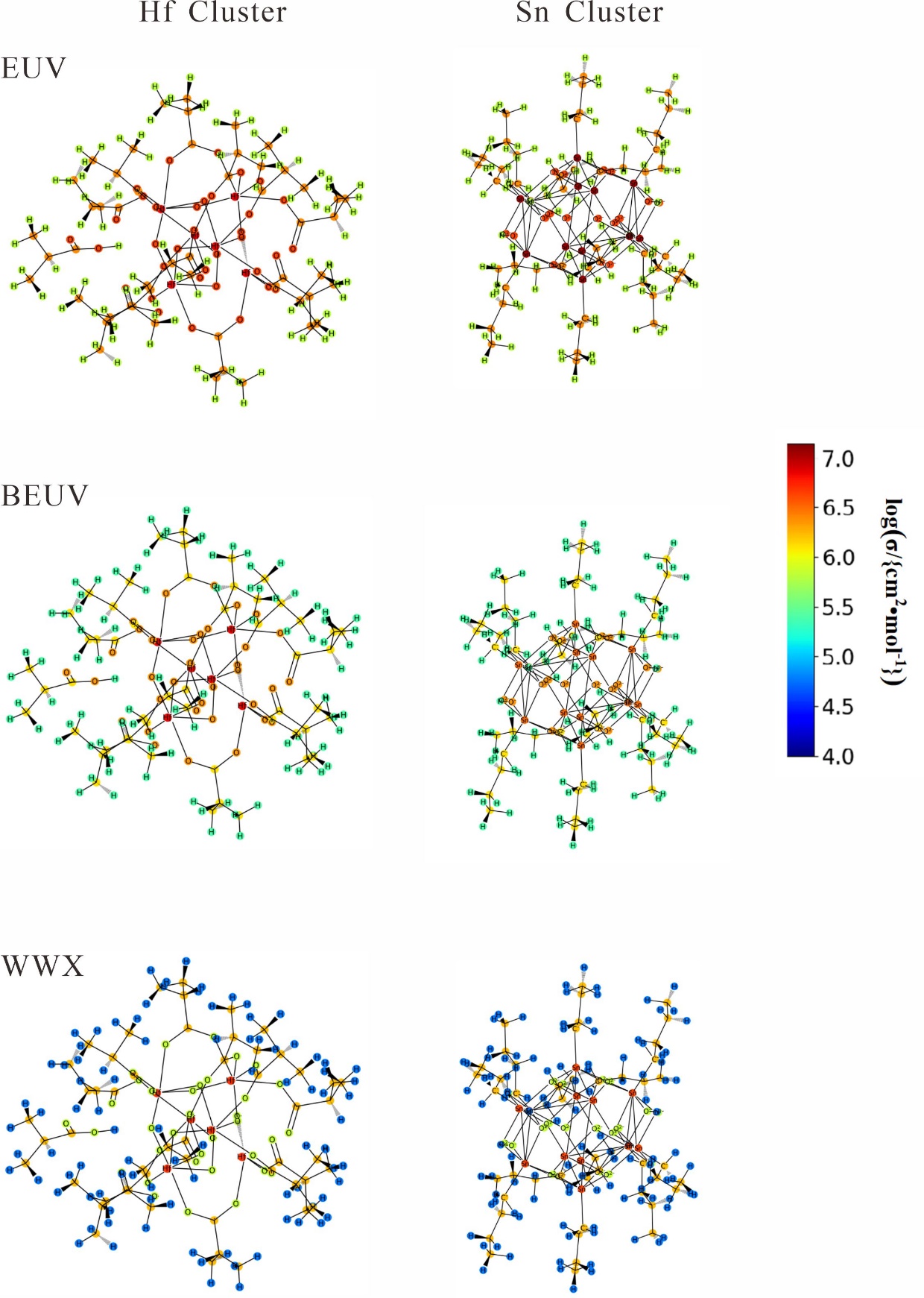


**Figure S1.** Metal-oxo cluster absorption cross section of Hf cluster and Sn cluster


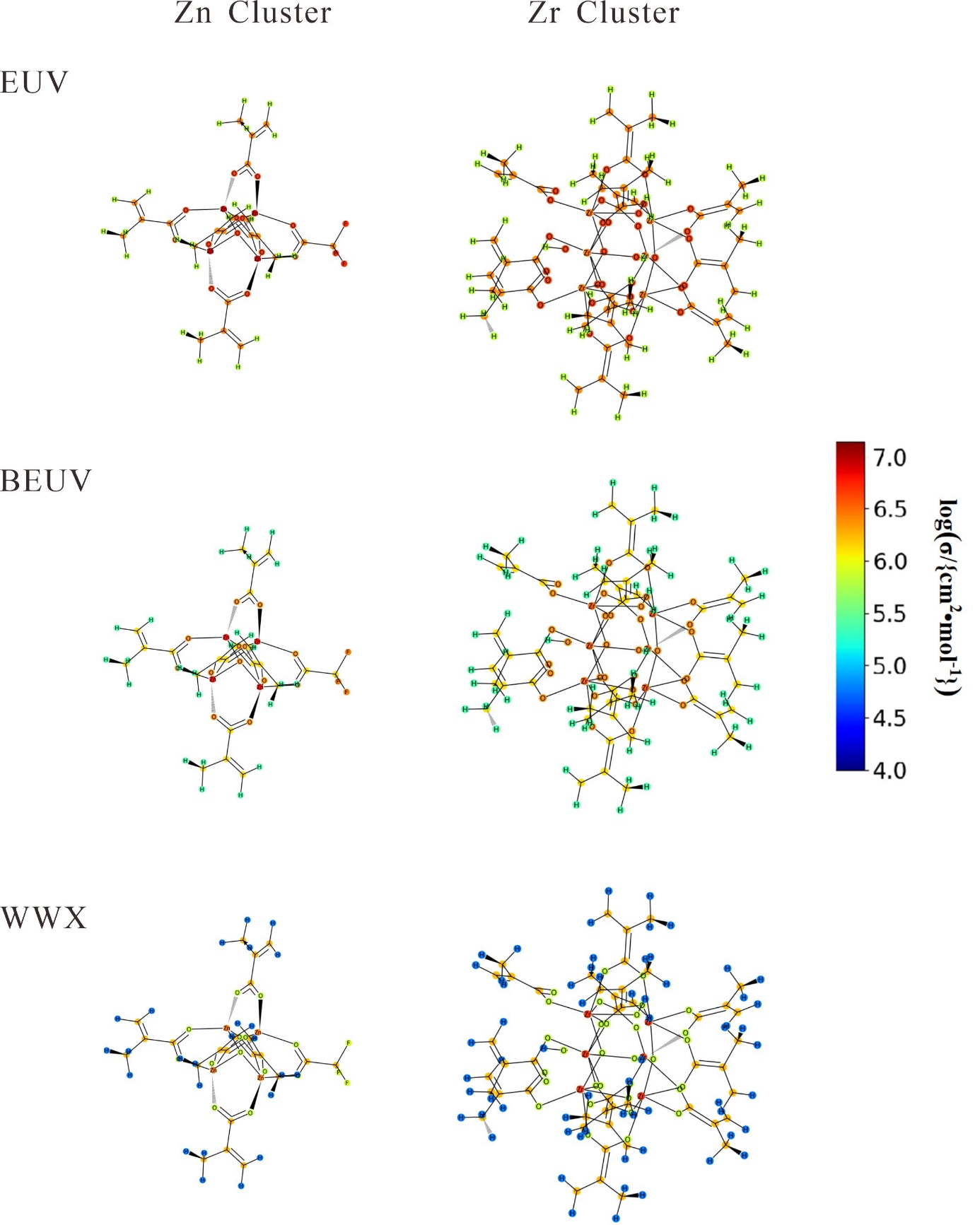


**Figure S2.** Metal-oxo cluster absorption cross section of Zn cluster and Zr cluster
